# Supplementary material for: Combined Model-Based Prediction for Non-Invasive Prenatal Screening
Source: Int J Mol Sci. 2022 Nov 30;23(23):14990. doi: 10.3390/ijms232314990 (PMC9737181; doi:10.3390/ijms232314990)
Supplement: Supplementary file 1 [file ijms-23-14990-s001.zip › ijms-2037718-supplementary.pdf]

Combined Model-Based Prediction for Non-Invasive Prenatal Screening

So-Yun Yang [1,†], Kyung Min Kang [2,†], Sook-Young Kim [3], Seo Young Lim [4], Hee Yeon Jang [1,2], Kirim Hong [5], Dong Hyun Cha [2,5], Sung Han Shim [1,2,*] and Je-Gun Joung [1,3,6*]

1 Department of Biomedical Science, College of Life Science, CHA University, Seongnam, Gyunggi-do 13488, Korea
2 Center for Genome Diagnostics, CHA Biotech Inc, Seoul 06135, Korea
3 CHA Future Medicine Research Institute, CHA Bundang Medical Center, Seongnam, Gyunggi-do 13520, Korea
4 Department of Biomedical Engineering, Hankuk University of Foreign Studies, Seoul, Korea
5 Department of Obstetrics and Gynecology, CHA Gangnam Medical Center, CHA University, Seoul 06135, Korea
6 Institute for Biomedical Informatics, CHA University School of Medicine, CHA University, Seongnam, Republic of Korea.
* Correspondence: e-mail@e-mail.com; Tel.:
† These authors contributed equally to this work

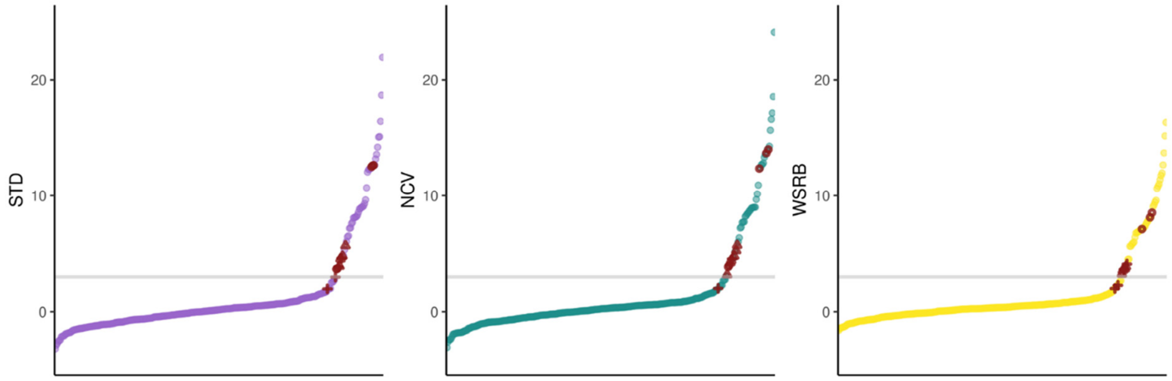

Figure S1. Z-scores of chromosomes 13, 18, and 21 calculated using three different methods.

Table S1. Number of trisomies predicted using three different methods.

	STD	NCV	WSRB	Confirmation
Trisomy 13	5	5	3	0
Trisomy 18	9	10	9	5
Trisomy 21	33	34	33	33
Total	47	49	45	38
